# YangXue QingNao Wan, a Compound Chinese Medicine, Attenuates Cerebrovascular Hyperpermeability and Neuron Injury in Spontaneously Hypertensive Rat: Effect and Mechanism

**DOI:** 10.3389/fphys.2019.01246

**Published:** 2019-10-01

**Authors:** Ying-Qian Jiao, Ping Huang, Li Yan, Kai Sun, Chun-Shui Pan, Quan Li, Jing-Yu Fan, Zhi-Zhong Ma, Jing-Yan Han

**Affiliations:** ^1^Department of Integration of Chinese and Western Medicine, School of Basic Medical Sciences, Peking University, Beijing, China; ^2^Tasly Microcirculation Research Center, Peking University Health Science Center, Beijing, China; ^3^Key Laboratory of Stasis and Phlegm, State Administration of Traditional Chinese Medicine of the People’s Republic of China, Beijing, China; ^4^Academy of Integration of Chinese and Western Medicine, Peking University Health Science Center, Beijing, China; ^5^Key Laboratory of Microcirculation, State Administration of Traditional Chinese Medicine of the People’s Republic of China, Beijing, China; ^6^State Key Laboratory of Core Technology in Innovative Chinese Medicine, Beijing, China

**Keywords:** hypertension, tight junction, caveolin-1, blood–brain barrier, ATP, microvessel

## Abstract

**Objective:**

The purpose of the study was to explore the effect of YangXue QingNao Wan (YXQNW), a compound Chinese medicine, on cerebrovascular hyperpermeability, neuronal injury, and related mechanisms in spontaneously hypertensive rat (SHR).

**Methods:**

Fourteen-week-old male SHR were used, with Wistar Kyoto (WKY) rats as control. YXQNW (0.5 g/kg/day), enalapril (EN, 8 mg/kg/day), and nifedipine (NF, 7.1 mg/kg/day) were administrated orally for 4 weeks. To assess the effects of the YXQNW on blood pressure, the systolic blood pressure (SBP), diastolic blood pressure (DBP), and mean blood pressure (MBP) were measured. After administering the drugs for 4 weeks, the cerebral blood flow (CBF), albumin leakage from microvessels in middle cerebral artery (MCA)-dominated area, and the number and morphology of microvessels were assessed in the hippocampus area and cortex. Neuronal damage and apoptosis were assessed by Nissl staining and TUNEL staining. To assess the mechanisms of cerebrovascular hyperpermeability, we performed immunofluorescence and Western blot to assess the expression and integrity of cerebral microvascular tight junction (TJ) and caveolin-1 (Cav-1) in cortex. Energy metabolism and Src-MLC-MLCK pathway in cortex were assessed then for elucidating the underlying mechanism of the observed effect of YXQNW.

**Results:**

Spontaneously hypertensive rat exhibited higher blood pressure, Evans blue (EB) extravasation, albumin leakage, increased brain water content, decreased CBF, perivascular edema, and neuronal apoptosis in the hippocampus and cortex, all of which were attenuated by YXQNW treatment. YXQNW inhibited the downregulation of TJ proteins, mitochondrial Complex I, Complex II, and Complex V, and upregulation of caveolin-1, inhibiting Src/MLCK/MLC signaling in SHR. YXQNW combined with EN + NF revealed a better effect for some outcomes compared with either YXQNW or EN + NF alone.

**Conclusion:**

The overall result shows the potential of YXQNW to attenuate blood–brain barrier (BBB) breakdown in SHR, which involves regulation of energy metabolism and Src/MLCK/MLC signaling. This result provides evidence supporting the application of YXQNW as an adjuvant management for hypertensive patients to prevent hypertensive encephalopathy.

## Introduction

Hypertension affects approximately 1.0 billion people in the world, with an incidence close to 30% (Marwick et al., 2015), which is a major risk factor for vascular diseases including coronary artery disease, congestive heart failure, stroke, end-stage renal disease, and peripheral vascular disease (Go et al., 2014).

The constriction or sclerosis of arteriole blood vessel can not only cause hypertension, but also impairs the microvascular integrity leading to plasma albumin leakage from cerebral vessels (Fan et al., 2015; Wang et al., 2018). Blood–brain barrier (BBB) disruption during acute and chronic hypertension has been noticed in animal models, which may contribute to hypertensive encephalopathy observed in human (Heistad, 1984). Although antihypertensive drugs can dilate arterioles and lower blood pressure (Chrysant, 2007), the effect of these drugs on BBB disruption during hypertension is largely unknown. On the other hand, at least some antihypertensive drugs have been reported as ineffective for prevention of BBB dysfunction (Mamo et al., 2017). Therefore, it is appealing to search for a management that possesses the potential to protect BBB dysfunction during hypertension thus benefits patients at risk of hypertensive encephalopathy.

YangXue QingNao Wan (YXQNW) is a compound Chinese medicine that contains the same herbs as Cerebralcare Granule (CG) does, but in different dosage form. Chinese herbs in YXQNW include Dang Gui (*Radix angelicae sinensis*), Bai Shao (*Radix paeoniae alba*), Chuan Xiong (*Rhizoma chuanxiong*), Ji Xue Teng (*Caulis spatholobi*), Di Huang (*Radix rehmanniae preparata*), Gou Teng (*Ramulus uncariae cum uncis*), Xia Ku Cao (*Spica prunellae*), Jue Ming Zi (*Semen cassiae*), Zhen Zhu Mu (*Concha margaritifera usta*), Yan Hu Suo (*Rhizoma corydalis yanhusuo*), and Xi Xin (*Herba asari*). YXQNW and CG have been used for the treatment of headache, dizziness, irritability, insomnia, and dreaminess in clinic (Huang et al., 2012). Previous studies confirmed the protective effect of CG on cerebrovascular hyperpermeability and neuron injury in rats and hamsters caused by ischemia and reperfusion (I/R) (Sun et al., 2010; Huang et al., 2012; Xu et al., 2015). However, it is not clear whether YXQNW can attenuate cerebrovascular hyperpermeability and neuron damage caused by hypertension. The present study aimed to explore the effect of YXQNW on BBB dysfunction during hypertension, and underlying mechanism in spontaneously hypertensive rats (SHRs).

## Materials and Methods

### Animals and Groups

The animals used in the study were SHR weighing 230–250 g with male Wistar Kyoto (WKY) rats weighing 220–240 g as control. The animals were housed in cages at 22 ± 2°C and a humidity of 40 ± 5% under a 12 h light/dark cycle, and received a standard diet and water *ad libitum*. The rats were fasted for 12 h before the experiment but allowed free access to water. The experimental procedures were carried out in accordance with the European commission guidelines (2010/63/EU). All rats were purchased from the Animal Center of Peking University Health Science Center. All animals were handled according to the guidelines of the Peking University Animal Research Committee. Experiment handling was approved by the Committee on the Ethics of Animal Experiments of Peking University Health Science Center (LA2017214).

Thirty WKY rats with mean blood pressure (MBP) of 90 ± 10 mm Hg were used in WKY + deionized water group (WKY + DW) as control; a total of 120 SHR (MBP, 140 ± 10 mm Hg) were included and randomly divided into four groups: SHR + deionized water group (SHR + DW), SHR + enalapril + nifedipine group (SHR + EN + NF), SHR + YXQNW group (SHR + YXQNW), and SHR + enalapril + nifedipine + YXQNW group (SHR + EN + NF + YXQNW).

The number of animals used in each group for determination of each parameter is detailed in Table 1.

**TABLE 1:** The number of animals for different experimental groups and various parameters.

	**Blood pressure**					
**Group**	**CBF and**	**Evans blue**	**Water content**	**Nissl stain, immunochemistry**	**Western blot**	**Total**
	**albumin leakage**			**and immunofluorescence**		
WKY + DW	8	6	6	4	6	30
SHR + DW	8	6	6	4	6	30
SHR + EN + NF	8	6	6	4	6	30
SHR + YXQNW	8	6	6	4	6	30
SHR + EN + NF + YXQNW	8	6	6	4	6	30
Total	40	30	30	20	30	150

*The same animals were used for detection of CBF and albumin leakage. The same animals were used for immunochemistry and immunofluorescence. The same animals were used for Western blot and ELISA. The 30 animals in each group were all subjected to evaluation of blood pressure. SHR + YXQNW: post-treatment with YXQNW at 500 mg/kg/day group. SHR + EN + NF: post-treatment with enalapril at 8 mg/kg/day + nifedipine at 7.1 mg/kg/day. WKY + DW: WKY given an equal amount of deionized water per day group; SHR + DW: SHR given an equal amount of deionized water per day group.*

### Drugs

YangXue QingNao Wan (batch number 160345) was provided by the Tasly Pharmaceutical Co. Ltd. (Tianjin, China). Enalapril (EN, batch number E6888) and nifedipine (NF, batch number N7634) were provided by the Sigma Chemical Company (St. Louis, MO, United States). YXQNW, EN, and NF were dissolved in DW to a concentration of 100, 1.6, and 1.4 mg/ml, respectively, before use.

### Drug Administration

The animals received each drug daily by gavage for 4 weeks at following doses: YXQNW at 500 mg/kg/day, EN at 8 mg/kg/day, and NF at 7.1 mg/kg/day. The animals in WKY + DW and SHR + DW groups were given an equal amount of deionized water. The dose of YXQNW used in the present study was determined based on our previous works (Xu et al., 2009; Sun et al., 2010). The doses of EN and NF were derived from the clinical use of human doses in the hypertension guidelines.

### Evaluation of Blood Pressure

Systolic blood pressure (SBP), diastolic blood pressure (DBP), and MBP of the rats were measured between 9 and 12 am on the day before administration, and 1, 2, 3, and 4 weeks after administration, respectively (*n* = 30). For this purpose, the rats were fixed in a net insulation cover under quiet and awake state for habituation with the temperature being preheated for 10 min at 37°C. SBP, DBP, and MBP were measured by intelligent non-invasive sphygmomanometer (U0130163, Softron Company, Japan), respectively, for three times, taking the average as the value at the time point (Tian et al., 2013).

### Cerebral Blood Flow Measurement

Cerebral blood flow (CBF) (*n* = 8) was measured using laser speckle perfusion image system (PeriScan PIM3 System; PERIMED, Stockholm, Sweden). In short, rats were anesthetized with pentobarbital sodium (0.1 g/kg body weight, i.p.), with an incision made through the scalp, and the skin was retracted to expose the skull. The periosteal connective tissue adherent to the skull was removed with a sterile cotton swab. A parietal bone window of 3 × 5 mm^2^ was opened with a hand-held drill on the right side 1 mm behind the coronal suture, and 1 mm lateral to sagittal suture as per described protocol (Xu et al., 2009). A low-powered He/Ne laser beam over the exposed parietal bone was directed by a computer-controlled optical scanner. The distance between the scanner head and cerebral cortex was 18.5 cm, with the scanner head parallel to the cerebral cortex surface. At each measuring site, the beam illuminated the tissue to a depth of 0.5 mm, as set in the instrument (Gu et al., 2018), and images were acquired after 10 min of basic observation. A color-coded image to denote specific relative perfusion level was displayed on a video monitor, and all images were evaluated with the software LDPI win 3.1 (PeriScan PIM3 System; PERIMED, Stockholm, Sweden), by which the number of perfusion unit for each image was calculated automatically.

### Observation of Microcirculation

Assessment of albumin leakage from cerebral venules was undertaken after 4 weeks of treatment. For this, rat was secured in a stereotactic frame and anesthetized with pentobarbital sodium (0.1 g/kg body weight, i.p.). A 3 × 5 mm^2^ cranial window was prepared as above at the same location, which corresponds to the margin of the middle cerebral artery (MCA) territory. The dura was removed and the pia mater was superfused continuously with 37°C warm physiological saline. The cerebral venules ranging from 35 to 45 μm in diameter and 200 μm in length were selected under a fluorescence microscope (X51WI, Olympus, Tokyo, Japan). Ten minutes before observation, the rat was intravenously infused with 50 mg/kg fluorescein isothiocyanate (FITC)-albumin (Sigma–Aldrich, St. Louis, MO, United States) through the femoral vein. Fluorescence signal (excitation wave length at 420–490 nm, emission wave length at 520 nm) was acquired using a super-sensitive CCD camera (USS-301, UNIQ Vision Inc., Santa Clara, CA, United States). The fluorescence intensities of FITC-albumin in the venules (v) and the perivenular interstitial area (i) were assessed with ImageJ (Bethesda, MD, United States) software. Albumin leakage was presented as i/v.

The adherent leukocytes were identified as cells that attached to the venular walls for >30 s. The fluorescence tracer Rhodamine 6G (Sigma, St. Louis, MO, United States) was administrated (5 mg/kg body weight) through femoral vein. After craniotomy, the cerebral cortex venules were observed under an upright intravital fluorescent microscopy (BX51WT; Olympus, Tokyo, Japan), illuminated with an argon laser beam with a wavelength of 543 nm (Wang et al., 2012).

### Evans Blue Leakage and Brain Water Content

Evans blue (EB) leakage was assessed as previously described (Wang et al., 2012) with some modifications (*n* = 6). In short, rats were anesthetized with pentobarbital sodium (0.1 g/kg body weight, i.p.), and EB dye (Sigma–Aldrich, St. Louis, MO, United States; 4%, 3 ml/kg) in saline was injected within 2 min into the left femoral vein and allowed to circulate for 3 h. Rats were transcardially perfused with phosphate-buffered saline (PBS) until colorless perfusion fluid drained from the right atrium. The amount of extravasated EB in the brain was determined by spectroflurophotometry at an excitation wavelength of 620 nm.

Brains (*n* = 6) were removed and quickly separated into the left and right cerebral hemispheres and weighed (wet weight). Brain specimens were then dried in an oven at 120°C for 48 h and weighed for determination of dry weight. The percentage of water content was presented as per [(wet weight−dry weight)/wet weight] × 100% (Wang et al., 2012).

### Nissl Staining and TUNEL Assay

Continuous sections starting from the optic chiasma were selected, and each section was 10 μm thick.

For Nissl staining (*n* = 4 for each group), the sections were stained with cresyl violet acetate (Sigma–Aldrich, St. Louis, MO, United States) and examined with light microscope (BX512DP70, Olympus, Tokyo, Japan) according to the standard procedure.

Transferase-mediated deoxyuridine triphosphate-biotin nick end labeling (TUNEL) assay was applied to assess the apoptotic neurons in CA1 region of hippocampus and cortex of rats, using the *in situ* cell death detection kit (Roche, United States), and conducted according to the instruction of the manufacturer. The number of total nuclei and TUNEL-positive nuclei in each field of CA1 region was scored with Image-Pro Plus 5.0 software, using laser confocal microscope (Axiovert200, Zeiss, Germany) with a 63× objective, and five fields of CA1 and cortex region were examined for each animal. As a control, a consecutive section was prepared and processed by the same procedure but without addition of TdT enzyme (Gu et al., 2018).

### Immunohistochemistry

For immunohistochemistry (*n* = 4 for each group), coronal fresh frozen sections were sliced starting from the optic chiasma in 10 μm thick using a cryostat (CM1800, Leica, Bensheim, Germany). The slicing method was the same as above. The sections were incubated with mouse anti-CD31 (1:50, Thermo Fisher Scientific, MA1-80069, Waltham, MA, United States) diluted in PBS overnight at 4°C after blocking with bovine serum albumin. Then the samples were incubated with a biotinylated secondary antibody followed by avidin–biotin–peroxidase complex. Positive staining was visualized with diaminobenzidine. The images were captured by a digital camera connected to a microscope (BX512DP70, Olympus, Tokyo, Japan). Five fields were randomly selected for each rat. The number of open microvessels and microvessels with perivascular edema was analyzed with Image-Pro plus 5.0 software (IPP, Media Cybernetic, Bethesda, MD, United States). Five fields of the hippocampus and cortex region were randomly selected and examined separately for each animal (Gu et al., 2018). The microvessels that had the CD31-positive endothelium sticking together without any lumen were defined as closed microvessels. The percentage of microvessels with perivascular edema and closed microvessels in each field was calculated.

### Immunofluorescence

For evaluation of the expression of tight junction (TJ) proteins claudin-5 and ZO-1 in cerebral microvessels, immunofluorescence staining was performed. The tissue preparation and staining was performed as per described protocol (Li et al., 2018), and the primary antibodies applied included: mouse anti-claudin-5 and anti-ZO-1 (1:50, Invitrogen, Camarillo, CA, United States), and rabbit anti-vWF (1:100, Millipore, Temecula, CA, United States). The brain sections were mounted, coverslipped, and photographed under a laser scanning confocal microscope (TCS SP5, Leica, Mannheim, Germany), as described previously (Li et al., 2018).

### Western Blot

Western blot analysis was conducted as described previously (Huang et al., 2012), using the tissue from the right neocortex of rats. Protein concentrations were estimated by the Bradford method, and an equal amount of protein (100 μg/lane) was diluted in 10× sample buffer, boiled, and loaded onto 12% SDS-PAGE gels and transferred to a nitrocellulose membrane (Hybond-C, Amersham Biosciences, United States) after separation by electrophoresis. Then, the membranes were incubated overnight at 4°C with the primary antibodies against GAPDH, occludin, ZO-1, myosin light chain (MLC), MLC kinase (MLCK), phospho-MLC (P-MLC) (Abcam, Cambridge, United Kingdom), claudin-5 (Santa Cruz Biotechnology, Santa Cruz, CA, United States), caveolin-1, Src, and P-Src (Tyr416) (Cell Signaling Technology, Beverly, MA, United States), ATP synthase-α subunit (ATP-α), ATP synthase-β subunit (ATP-β) (Thermo Fisher Scientific, Waltham, MA, United States), and ATP-5D (Abcam, Cambridge, United Kingdom). The membranes were washed with tris-buffered saline and tween20 (TBST) and incubated with the respective horseradish peroxidase-conjugated secondary antibodies at a 1:5000 dilution for 60 min at room temperature. The protein bands were detected by enhanced chemiluminescence, and the band intensities were quantified by densitometry and expressed as mean area density using the ImageJ (Bethesda, MD, United States) software.

### ELISA

The rats were perfused with NS, and a tissue block of about 2 mm^3^ was removed from the cortex for the assessment of the concentrations of Complex I, Complex II, Complex IV, Complex V, ATP, AMP, and ADP by ELISA kits (Abcam, Cambridge, United Kingdom) according to the manufacturer’s protocol. OD values were determined by enzyme microplate reader (Thermo Multiskan Mk3, Thermo Fisher Scientific Inc., Barrington, IL, United States). The concentrations of Complex I, Complex II, Complex IV, Complex V, ATP, AMP, ATP/ADP, and ATP/AMP were calculated based on the standard curves (Chen et al., 2018).

### Statistical Analysis

The data of all groups were expressed as means ± SEM. Meanwhile, statistical analysis was conducted by one-way or two-way analysis of variance, followed by Bonferroni test. A value of *P* < 0.05 was considered statistically significant.

## Results

### YXQNW Attenuates the Blood Pressure and Cerebral Blood Flow Decrease in SHR

**FIGURE 1:**
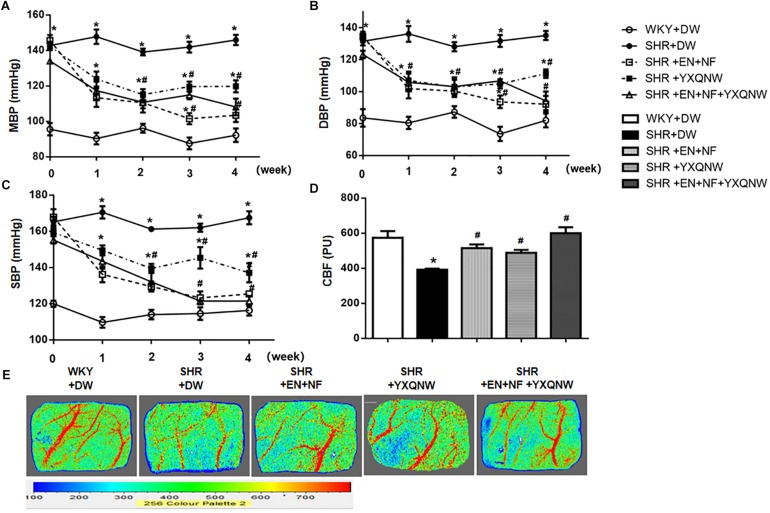
YXQNW attenuates the blood pressure and cerebral blood flow decrease in SHR. Panels **(A–C)** show the change of MBP, DBP, and SBP with time, respectively, before (0 week) and after (1, 2, 3, and 4 week) drug administration in each group. *n* = 30. **(D)** Quantitative analysis of CBF in different groups. The unit of *Y*-axis is perfusion unit (PU). **(E)** The representative images of CBF of ipsilateral cortex in different groups. The magnitude of CBF is represented by different colors, with blue to red denoting low to high. Values are the mean ± SEM. *^∗^P* < 0.05 vs. control group, *^#^P* < 0.05 vs. SHR + DW group.

The blood pressure of animals was recorded over time. As shown in Figures 1A–C, before administration, MBP, DBP, and SBP in the SHR groups were significantly higher than that in the control group. Treatment with either EN + NF or YXQNW or EN + NF + YXQNW reduced the MBP, DBP, and SBP in SHR gradually with time, reaching a value close to WKY group at 4 weeks with YXQNW alone treatment being less effective.

Cerebral blood flow was determined at the end of experiment and the representative images of the five groups are shown in Figure 1E, while the quantification of the results is presented in Figure 1D. Compared with control group (573.7 ± 37.64), CBF in SHR + DW group decreased significantly (391.8 ± 6.213), while EN + NF, YXQNW, and EN + NF + YXQNW administration markedly relieved CBF of SHR (515.3 ± 20.38 in EN + NF group, 488.3 ± 16.74 in YXQNW group, 599.5 ± 33.99 in EN + NF + YXQNW group, *P* < 0.05).

### YXQNW Increases the Number of Closed Capillaries, Inhibits Perivascular Edema in the Hippocampus and Cortex of SHR

**FIGURE 2:**
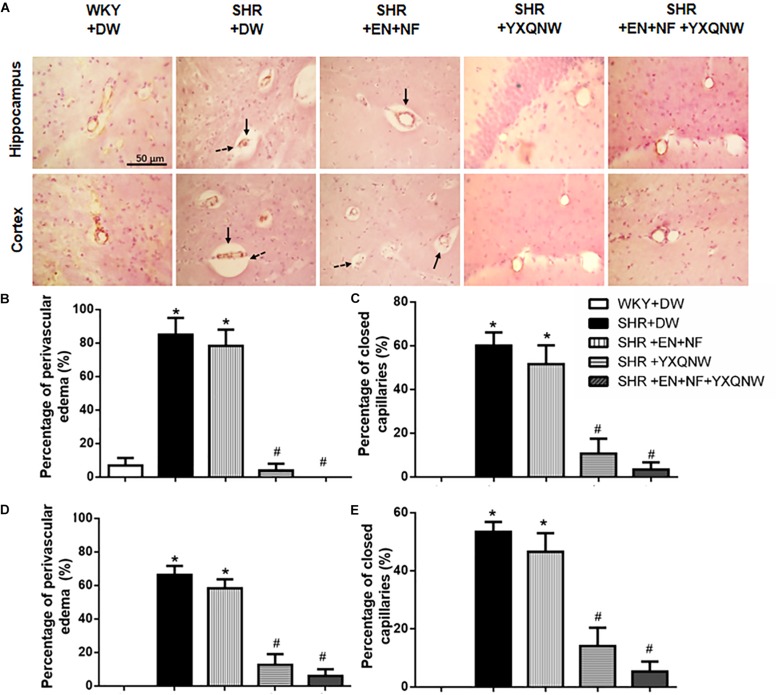
YXQNW inhibits the perivascular edema and reduces the number of closed capillaries in the hippocampus and cortex of SHR. **(A)** Representative images in the region between CA1 to DG of hippocampus and region of cortex stained by immunohistochemistry staining for CD31 in all the groups. Dotted arrows stand for closed capillaries and solid arrows stand for blood vessels with perivascular edema. Bar = 50 μm. **(B)** The percentage of the capillaries with perivascular edema to the total number of capillaries in a region between CA1 and DG of hippocampus evaluated in five randomly selected micrographs for each animal. **(C)** The percentage of the closed capillaries to the total number of capillaries in hippocampus evaluated in five randomly selected micrographs for each animal. **(D)** The percentage of the capillaries with perivascular edema to the total number of capillaries in cortex evaluated in five randomly selected micrographs for each animal. **(E)** The percentage of the closed capillaries to the total number of capillaries in cortex evaluated in five randomly selected micrographs for each animal. *^∗^P* < 0.05 vs. control group, *^#^P* < 0.05 vs. SHR + DW group. *n* = 20.

Displayed in Figure 2A are the representative images of hippocampus and cortex region acquired by immunohistochemistry staining for CD31 in all the groups, with the statistic result for the number of closed microvessels (dotted arrows) and the microvessels with perivascular edema (solid arrows) in each group presenting in Figures 2B–E. Apparently, in the hippocampus, compared to control group, the percentage of closed capillaries in SHR + DW group was significantly higher (60.00 ± 6.12 vs. 0.00 ± 0.00, *P* < 0.05). Interestingly, YXQNW and EN + NF + YXQNW treatment attenuated this alteration (10.67 ± 6.86 and 3.33 ± 3.33, *P* < 0.05), while treatment with EN + NF did not show significant effect (51.60 ± 8.60) (Figure 2C). Similar results were observed in the cortex (*P* < 0.05), as presented in Figure 2E. The quantitative evaluation of the percentage of microvessels with perivascular edema in hippocampus (Figure 2B) and cortex (Figure 2D) revealed similar results (*P* < 0.05). These results offered morphological evidence for the ability of the drugs to protect microvessels from collapsing in SHR.

### YXQNW Protects BBB From Breakdown in SHR

**FIGURE 3:**
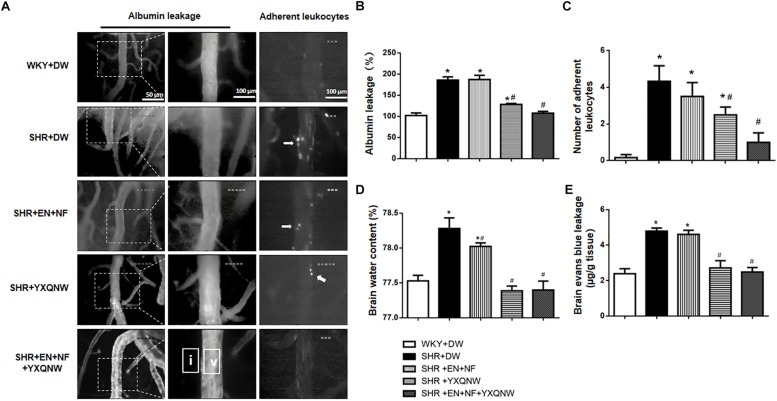
YXQNW reduces venous albumin leakage, adherent leukocytes, Evans blue extravasation, and brain water content in SHR. **(A)** Representative images for albumin leakage from venules and adherent leukocytes in venules in all groups. Rectangles representing the areas for determination of fluorescence arrows stand for adherent leukocytes. Bar = 50 μm. v, cerebral venule; i, interstitial tissue. **(B)** Statistic analysis of albumin leakage. **(C)** Statistic analysis of adherent leukocytes. **(D)** The quantitative analysis of brain water content. **(E)** The quantitative analysis of Evans blue leakage. Values are the mean ± SEM. *^∗^P* < 0.05 vs. control group, *^#^P* < 0.05 vs. SHR + DW group.

The ameliorating effect of YXQNW on the perivascular edema suggests its potential to protect BBB. We thus assessed the effect of EN + NF, YXQNW, and their combination on BBB permeability by albumin leakage and EB dye extravasation at the right cerebral hemisphere of rats in various groups. As shown in Figures 3A–C, compared with the WKY + DW group (102.0 ± 5.95), albumin leakage in SHR + DW group was markedly increased (185.8 ± 7.68), which was not attenuated by EN + NF alone (187.1 ± 10.06) but by YXQNW and EN + NF + YXQNW treatment (128.3 ± 2.22 and 107.6 ± 4.06) (Figures 3A,B). Compared with the WKY + DW (0.17 ± 0.17), the leukocyte adhesion in SHR group increased significantly (4.33 ± 0.84), which was not attenuated by EN + NF (3.50 ± 0.76, *P* > 0.05). In contrast, YXQNW and EN + NF + YXQNW treatment attenuated the leukocyte adhesion significantly, reaching to 2.50 ± 0.43 and 1.00 ± 0.52, respectively (Figures 3A,C).

The amount of EB dye extravasation in the right cerebral hemisphere was markedly increased in the SHR group (4.79 ± 0.17) compared with the WKY group (2.39 ± 0.28) (Figure 3E), which was not affected by EN + NF (4.60 ± 0.23, *P* > 0.05). Whereas, YXQNW and EN + NF + YXQNW treatment attenuated the EB dye extravasation significantly, reaching to 2.71 ± 0.41 and 2.48 ± 0.25, respectively (Figure 3E). Likewise, the brain water content varied among groups in a similar fashion (Figure 3D), pointing toward a conclusion that hypertension impairs cerebral microvessels leading to BBB hyperpermeability, which was attenuated by YXQNW but not by EN + NF.

### YXQNW Protects the Neurons in CA1, CA2, CA3, and DG Regions of Hippocampus

**FIGURE 4:**
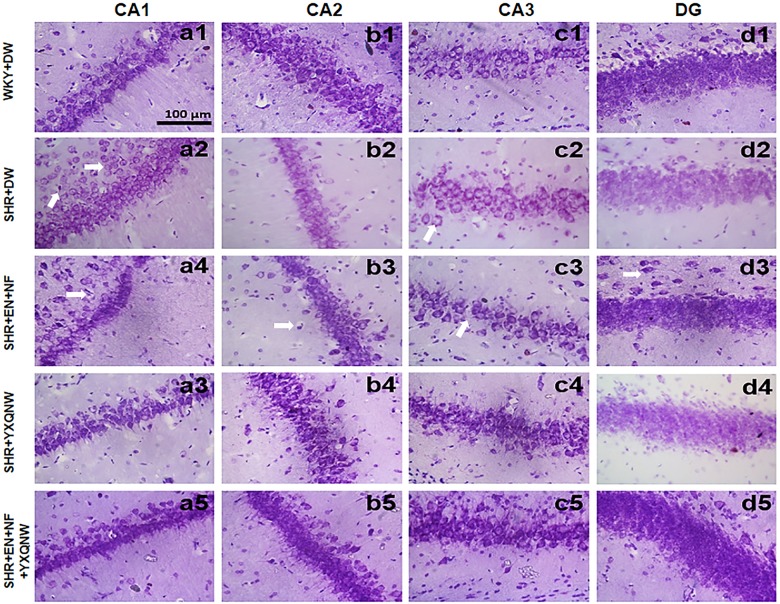
YXQNW protects the neuron structure in CA1, CA2, CA3, and DG regions. Representative images of neuron structure in CA1, CA2, CA3, and DG regions in hippocampus of WKY + DW group **(a1–d1)**, SHR + DW group **(a2–d2)**, EN + NF group **(a3–d3)**, YXQNW group **(a4–d4)**, and EN + NF + YXQNW group **(a5–d5)**. Bar = 100 μm.

To further specify the effect of YXQNW and EN + NF on the neurons of SHR, TUNEL and Nissl assays were performed for the hippocampus. Figure 4 shows the representative images of Nissl staining in various groups. In the CA1, CA2, CA3, and DG regions of hippocampus, neurons of control group showed normal morphological features, while in SHR + DW group diverse neuronal damages such as scattered permutations and nuclear pyknosis occurred. Compared with SHR + DW group, YXQNW and EN + NF + YXQNW treatment effectively prevented the neuronal damages in SHR, while EN + NF did not show an obvious effect.

**FIGURE 5:**
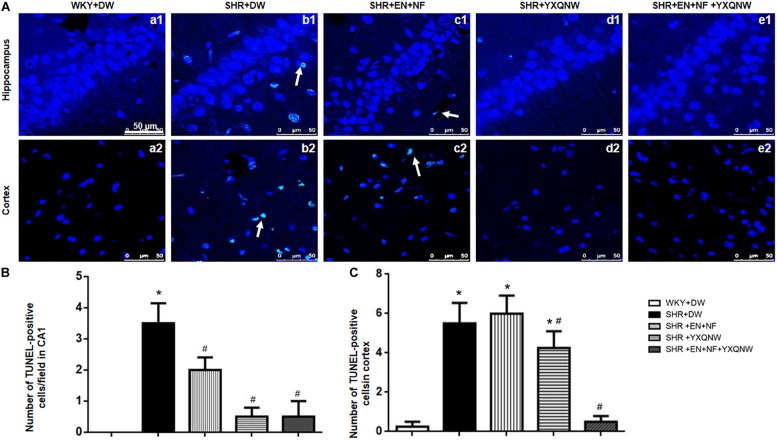
YXQNW inhibits CA1 neuron death in hippocampus and cortex. **(A)** Representative images of neuron death in hippocampus CA1 and cortex of WKY + DW group **(a1,a2)**, SHR + DW group **(b1,b2)**, EN + NF group **(c1,c2)**, YXQNW group **(d1,d2)**, and EN + NF + YXQNW group **(e1,e2)**. White arrows indicate the localization of TUNEL-positive cells. Bar = 50 μm. **(B)** Statistic analysis of neuron death, the data were derived from five randomly selected micrographs in hippocampus for each animal. **(C)** Statistic analysis of neuron death evaluated in five randomly selected micrographs in cortex for each animal. The area of each micrograph selected was 2.1 × 10^4^ μm^2^. Values are the mean ± SEM. *^∗^P* < 0.05 vs. control group, *^#^P* < 0.05 vs. SHR + DW group. *n* = 20.

Figure 5 shows a large number of TUNEL-positive cells in the CA1 region and cortex region of SHR, which were hardly observed in the control group (0.00 ± 0.00). The high blood pressure-evoked increase in the number of TUNEL-positive cells in CA1 region [Figures 5A(a1–e1),B] (3.50 ± 0.65) and cortex region (5.50 ± 1.04) [Figures 5A(a2–e2),C] was significantly reduced in YXQNW-treated group (CA1: 0.50 ± 0.23, cortex: 4.25 ± 0.85, *P* < 0.05) and EN + NF + YXQNW-treated group (CA1: 0.50 ± 0.50, cortex: 0.50 ± 0.29, *P* < 0.05). Quantitative evaluation of TUNEL-positive cells revealed that EN + NF treatment reduced the number of TUNEL-positive cells in CA1 region as well, although less effective than YXQNW and EN + NF + YXQNW treatment (Figure 5B). Whereas, in cortex, the three treatments manifested differently as to the effect on the number of TUNEL-positive cells with EN + NF having no effect (6.00 ± 0.91, *P* > 0.05), while EN + NF + YXQNW being more effective than YXQNW alone (0.50 ± 0.29 vs. 4.25 ± 0.85, *P* < 0.05) (Figure 5C).

### YXQNW Maintains the Integrity of Endothelial Cell Junctions

**FIGURE 6:**
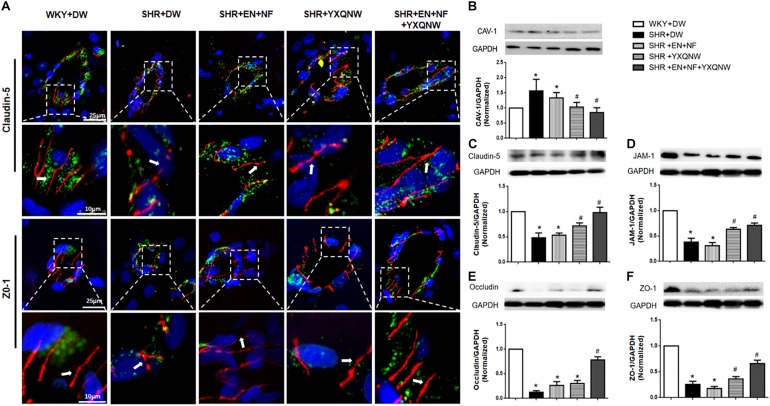
YXQNW protects the decrease in TJ proteins and caveolin-1 expression. **(A)** Representative immunofluorescence confocal images of claudin-5 and ZO-1. Claudin-5 (red) and ZO-1 (red) localized at the peripheral of endothelial cells with marker vWF (green). Note the continuous distribution of claudin-5 and ZO-1 in cortical microvessels of control group. The disruption of claudin-5 and ZO-1 staining in SHR + DW was not reduced in the SHR + EN + NF group but markedly reduced in the YXQNW treatment group and EN + NF + YXQNW treatment group. White arrows indicate the localization of claudin-5 or ZO-1. **(B)** Representative Western blots and quantitative analysis of caveolin-1. **(C)** Representative Western blots and quantitative analysis of claudin-5. **(D)** Representative Western blots and quantitative analysis of JAM-1. **(E)** Representative Western blots and quantitative analysis of occludin. **(F)** Representative Western blots and quantitative analysis of ZO-1. Values are the mean ± SEM. *^∗^P* < 0.05 vs. control group, *^#^P* < 0.05 vs. SHR + DW group.

Endothelial cell TJ is an essential contributor to BBB. To gain insight into the rationale behind the role of YXQNW in maintaining BBB integrity, vascular endothelial cell TJ proteins claudin-5 and ZO-1 were examined for various groups by confocal microscopy (Figure 6A) revealing that claudin-5 localized between endothelial cells as continuous lines in control group. In SHR + DW group, these continuous distributions were disrupted apparently, becoming dotted lines, concomitant with reduction in immune staining, indicating a decrease in claudin-5 expression. This decrease was not restored by EN + NF treatment but alleviated evidently by YXQNW treatment and YXQNW combined with EN and NF treatment. These results were confirmed by Western blot (Figure 6C). Similar results were obtained for ZO-1 (*P* < 0.05, *n* = 4) (Figures 6A,F), JAM-1 (*P* < 0.05, *n* = 4) (Figure 6D), and occludin (*P* < 0.05, *n* = 4) (Figure 6E). These results indicated that YXQNW could alleviate the reduction in TJ proteins expression of SHR, which may account for the protective role of the YXQNW in BBB breakdown.

Western blot was used to analyze the expression of caveolin-1, another determinant of vascular permeability (Figure 6B), showing a marked increase in caveolin-1 protein level in the SHR + DW group compared with control group (1.71 ± 0.37 vs. 1.00 ± 0.00). This increase was not obviously attenuated by EN + NF treatment (1.35 ± 0.22), but protected by YXQNW treatment and EN + NF + YXQNW treatment with the later exhibiting a better effect (1.05 ± 0.15 and 0.90 ± 0.15, *P* < 0.05, *n* = 6), which may confirm the protective role of the drugs in BBB breakdown from transcellular pathways.

### YXQNW Attenuates Energy Metabolism Disturbance

**FIGURE 7:**
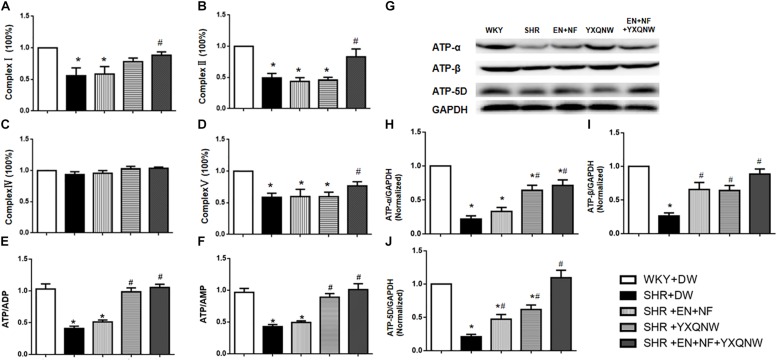
YXQNW increases the expression of Complex I, Complex II, and Complex V and elevates the value of ATP/ADP, ATP/AMP, and the expression of ATP-α, ATP-β and ATP-5D in the cerebral cortex. **(A–D)** Statistical results from ELISA for Complex I, Complex II, Complex IV, and Complex V (*n* = 6), respectively. **(E,F)** Statistical results from ELISA for the value of ATP/ADP and ATP/AMP (*n* = 6). **(G)** Representative Western blots of ATP-α, ATP-β, and ATP-5D. **(H–J)** Quantitative analysis of ATP-α, ATP-β, and ATP-5D. *^∗^P* < 0.05 vs. control group, *^#^P* < 0.05 vs. SHR + DW group.

We next assessed the energy metabolism in different conditions, which is closely related to mitochondria function and plays a critical role in maintaining BBB integrity. ELISA analysis revealed a decrease in the content of Complexes I, II, and V in SHR compared with control group (*P* < 0.05, *n* = 6). Treatment with EN + NF + YXQNW attenuated this decrease, while either EN + NF or YXQW alone had no effect (Figures 7A,B,D, *P* > 0.05, *n* = 6). Meanwhile, there was no significant difference in the content of Complex IV between the five groups (Figure 7C). Correspondingly, ATP/ADP and ATP/AMP diminished in SHR, which was ameliorated by YXQNW alone and EN + NF + YXQNW (Figures 7E,F, *P* < 0.05, *n* = 6). Western blot was further performed to assess the expression of ATP-α, ATP-β, and ATP-5D, the three subunits of Complex V, in different groups (Figure 7G). The result showed that all the three proteins decreased in SHR (Figures 7H–J, *P* < 0.05, *n* = 6), implying impairment of ATP synthases. This decrease was attenuated by treatment with EN + NF or/and YXQNW and the combination of the two with EN + NF + YXQNW being the most effective (*P* < 0.05, *n* = 6).

### YXQNW Inhibits Src/MLCK/MLC Signaling Pathway

**FIGURE 8:**
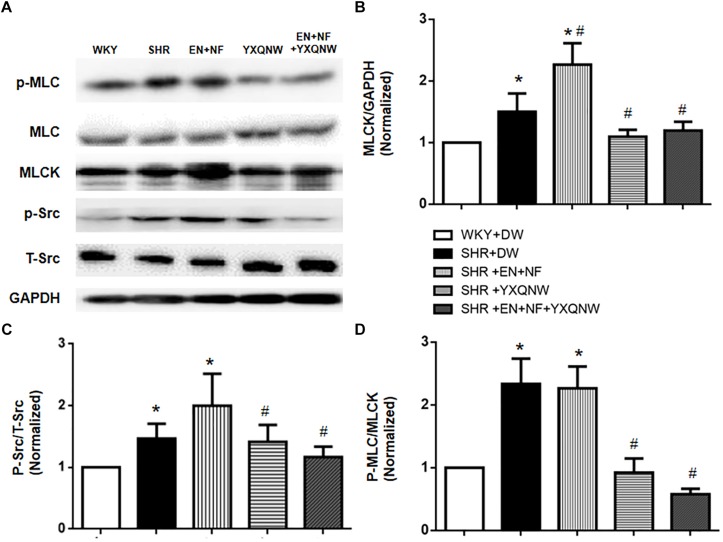
YXQNW inhibits Src/MLCK/MLC signaling pathway of SHR. **(A)** Representative Western blots of p-Src, MLCK, and p-MLC. **(B–D)** Quantitative analysis of p-MLC, MLCK, and p-Src, respectively. Values are the mean ± SEM. *^∗^P* < 0.05 vs. control group, *^#^P* < 0.05 vs. SHR + DW group.

Activation of MLCK by phosphorylated Src kinase has been shown to lead to increased actin–myosin interaction and subsequently increased paracellular permeability (Birukov et al., 2001). We thus determined the expression of proteins involved in Src/MLCK/MLC signaling pathway by Western blot in different conditions (Figure 8A). The result shows that as compared with control, SHR exhibited a significant increase in the expression of MLCK (1.49 ± 0.25 vs. 1.00 ± 0.00), p-Src/T-Src (1.49 ± 0.25 vs. 1.00 ± 0.00), and p-MLC/T-MLC (2.42 ± 0.38 vs. 1.00 ± 0.00), suggesting an increased microvascular permeability via paracellular pathway. Treatment with EN + NF had no effect on the elevated expression of p-Src and p-MLC (*P* > 0.05, *n* = 6) (Figures 8C,D), even further enhanced the increase of MLCK (2.33 ± 0.37) (Figure 8B), suggesting ineffectiveness of EN + NF in modulating Src/MLCK/MLC signaling. On the other hand, YXQNW alone or its combination with EN + NF significantly ameliorated the increase in the expression of the three proteins (MLCK: 1.11 ± 0.12 and 1.19 ± 0.14, respectively; p-Src/T-Src: 1.44 ± 0.26 and 1.20 ± 0.15, respectively; and p-MLC/T-MLC: 0.95 ± 0.27 and 0.62 ± 0.05, respectively) (Figures 8B–D), highlighting the implication of Src/MLCK/MLC signaling in the protective effect of YXQNW on BBB breakdown in SHR.

## Discussion

The dysfunction of BBB in SHR has been known for >40 years (Hazama et al., 1975; Ueno et al., 2004). Consistent with these results, we observed in the present study an increased permeability of BBB in SHR, manifesting enhanced albumin leakage from cerebral venules and EB extravasation in the brain as well as increased brain water content. Importantly, this BBB breakdown cannot be prevented by treatment with EN plus NF, the two commonly used antihypertensive drugs, but rather protected by YXQNW. This result highlights YXQNW as a prophylaxis for the patients at risk of hypertensive encephalopathy.

As an animal model prone to BBB dysfunction, SHR has been used to explore the pathogenesis of BBB disruption during chronic hypertension (Seyed et al., 2012). Several mechanisms have been reported to mediate the hypertension-caused BBB disruption, including enhanced transendothelial cell transport (Tagami et al., 1983), structure change in TJ and altered distribution of glucose transporter-1 (Lippoldt et al., 2000), increased expression of AQP4 in the end feet of astrocytes (Ishida et al., 2006), and the like. The present study revealed that compared with WKY rats, SHR presented a decreased expression of TJ proteins and an increased expression of caveolin-1, the principal protein of caveolae. This result suggests that both paracellular and transcellular pathways are implicated in the BBB dysfunction of SHR. In line with the result of enhanced BBB permeability, the dysregulated expression of TJ proteins and caveolin-1 in SHR was attenuated by YXQNW but not by EN + NF. Interestingly, supplement of EN + NF potentiated the effect of YXQNW to relive the dysregulation of caveolin-1 and some of the TJ proteins, such as in the case of claudin-5, occludin, and ZO-1, implying that some coordination between YXQNW and antihypertensive drugs likely occurs.

The present study revealed an impaired energy metabolism in SHR, possibly due to sclerosis and spasm of the arteries in SHR brain which lead to insufficient perfusion, causing temporary brain tissue hypoxia (Garrido and Griendling, 2009) which impacts the structure and function of mitochondria. The impaired energy metabolism may result in mar functional cell skeleton, including dissembling of F-actin and improperly regulated myosin–actin interaction, since both of which are energy-dependent process (Hinshaw et al., 1993). It is worth noting that a normally functional cell skeleton is known to provide a support for the integrity of endothelial cell junctions and is thus involved in regulation of vascular permeability (Alexander et al., 2015). In the present study, YXQNW, but not EN + NF, restored the impaired energy metabolism in SHR, which at least partially contributes to the protective effect of YXQNW on BBB breakdown.

Increasing evidence indicates the importance of Src signaling in modulation of BBB. Src activation impacts interaction between myosin and actin filaments through a pathway involving MLCK and MLC, leading to dysregulation of cell junction proteins (Guerrero et al., 2004). In addition, Src activation evokes phosphorylation of caveolin-1 (Labrecque et al., 2004), which not only provokes *trans*-endothelial pathway but also causes degradation of junction proteins, collectively resulting in BBB breakdown. The present study observed an increase in p-Src, MLCK, and p-MLC in SHR, suggesting implication of this signaling in BBB hyperpermeability. The reason for the activation of Src in SHR is at present unknown. One likelihood is oxidative stress generated from disrupted respiratory chain as evidenced by downregulation of Complexes I, II, and V. Impressively, YXQNW, but not EN + NF, inhibited the activation of Src/MLCK/MLC-signaling pathway, suggesting involvement of this signaling in the protective effect of YXQNW on BBB breakdown in SHR. Nevertheless, more study is needed to explore the possible involvement of other mechanism in the BBB dysfunction in SHR and the role of YXQNW in the occasion concerned.

Contracted arteriole in hypertensive patients reduces the blood supply to the irrigated brain territory. In addition, following BBB breakdown, albumin leakage and perivascular edema occurs, which imposes pressure on microvessels leading to narrowing even closing of the vessels, as evidenced in our study. As a consequence, the neurons will be subjected to deprivation of oxygen and glucose, which provokes oxidative stress resulting in neuron apoptosis. As expected, we observed a significantly increased apoptotic neurons in SHR, which may probably underlie hypertensive dementia. Administration of EN + NF reduced the number of TUNEL-positive cells in CA1, but had no effect on that in cortex. On the contrary, administration of YXQNW diminished TUNEL-positive neurons in both CA1 and cortex significantly, suggesting broader effect of this medicine than that of EN + NF in prevention of hypertension-related encephalopathy.

As a compound Chinese medicine, YXQNQ consists of 11 herbs with each containing numerous bioactive ingredients. Identification of the ingredient(s) in YXQNW responsible for the effects observed in the present study is a huge task, which needs much more work in the future. Moreover, the present study did not evaluate the intravascular surface and intravascular water, which may affect the brain wet weight thus contributes to W/D. This issue needs to be investigated by further study. Nevertheless, this limitation does not influence the conclusion that BBB is impaired in SHR thanks to the other evidence such as albumin leakage from cerebral venules and EB extravasation.

## Conclusion

In conclusion, the present study demonstrated the potential of YXQNW to attenuate BBB breakdown in SHR without interfering with the antihypertensive effect of EN + NF, which involves regulation of energy metabolism and Src/MLCK/MLC signaling. This result suggests YXQNW as an adjuvant management for hypertensive-adjective patients to prevent hypertensive encephalopathy.

## Data Availability Statement

All datasets generated for this study are included in the manuscript/supplementary files.

## Ethics Statement

The experiment handling was approved by the Committee on the Ethics of Animal Experiments of Peking University Health Science Center (LA2017214).

## Author Contributions

Y-QJ performed the research, analyzed the data, and wrote the manuscript. PH and KS contributed to the animal experiments. LY contributed to the Nissl stain, immunohistochemistry, and immunofluorescence. C-SP and PH contributed to the Western blotting. QL contributed to the other experiments. J-YF revised the manuscript. Z-ZM and J-YH designed and funded the research, interpreted the data, and finally approved the submission of this manuscript. All authors read and agreed with the manuscript.

## Conflict of Interest

The authors declare that the research was conducted in the absence of any commercial or financial relationships that could be construed as a potential conflict of interest.
